# Dengue Virus Type 3 Infection in Traveler Returning from West Africa

**DOI:** 10.3201/eid1511.081736

**Published:** 2009-11

**Authors:** Laetitia Ninove, Philippe Parola, Cécile Baronti, Xavier De Lamballerie, Philippe Gautret, Barbara Doudier, Rémi N. Charrel

**Affiliations:** Fédération de Microbiologie Clinique Assistance Publique Hôpitaux de Marseille, Marseille, France

**Keywords:** Dengue, West Africa, Côte d’Ivoire, surveillance, traveler, viruses, letter

**To the Editor:** GeoSentinel, the global surveillance program of the International Society of Travel Medicine, recently reported that returning travelers may serve as sentinels for local outbreaks of dengue fever in tropical areas to which it is endemic (*1*). We investigated a case of dengue virus (DENV) type 3 (DENV-3) infection in a traveler returning to France from West Africa, which provided evidence for DENV-3 circulation in Côte d’Ivoire.

A 53-year-old French expatriate living in Abidjan, the economic capital of Côte d’Ivoire, arrived in France on July 17, 2008, and was hospitalized 4 days later with fever of 40°C, headache, asthenia, anorexia, chills, diffuse arthralgia, and myalgia. Results of a physical examination were normal except for a diffuse nonpetechial macular rash and moderate hepatosplenomegaly. A tourniquet test was not performed. At admission, platelet count was 103 cells/mm^3^ and leukocyte count was 2,410 cells/mm^3^. Thin and thick blood smears and results of the immunochromatographic test (Binax NOW malaria tests; Binax, Portland, ME, USA) were negative. The patient recovered without sequelae and was discharged 6 days after admission.

At admission, chikungunya virus–specific immunoglobulin (Ig) G and IgM were not detected by indirect immunofluorescence tests (*2*). IgG, but not IgM, specific for DENV was detected by an immunochromatic test (Panbio Dengue Duo Cassette; Biotrin, Lyon, France) and confirmed by ELISA (PanBio dengue duo test). However, DENV RNA was demonstrated in serum by using 4 reverse-transcription–PCR (RT-PCR)–based assays: a positive result in a flavivirus universal assay (*3*), a positive result in a DENV-1–4 real-time RT-PCR (*4*), a positive result in a DENV-3–specific RT-PCR, and a negative result in a specific RT-PCR for DENV-1, -2, and -4 (*4*). The concomitant finding of DENV RNA and IgG against DENV suggests the patient had dengue infection before this episode.

DENV-3 viremia was confirmed by sequencing a 547-nt region of the envelope gene (GenBank accession no. FJ587232, nt 852–1398 referring to the H87 DENV-3 prototype strain). Our sequence aligned with homologous DENV-3 sequences retrieved from GenBank and used for phylogenetic analysis (Figure). Our patient, in whom classic dengue fever was diagnosed, was infected with a strain that belonged to genotype III most closely related to strains isolated in Singapore, Taiwan, Sri Lanka, India, and Saudi Arabia. Its closest relatives were the strain from Saudi Arabia isolated in 2004 and another strain claimed in ProMED by Japanese researchers to have been isolated in 2008 from a Japanese traveler returning from Côte d’Ivoire (*6*). Therefore, this strain is likely to have originated in the Middle East, the Indian subcontinent, or Southeast Asia rather than in Central or South America.

**Figure:**
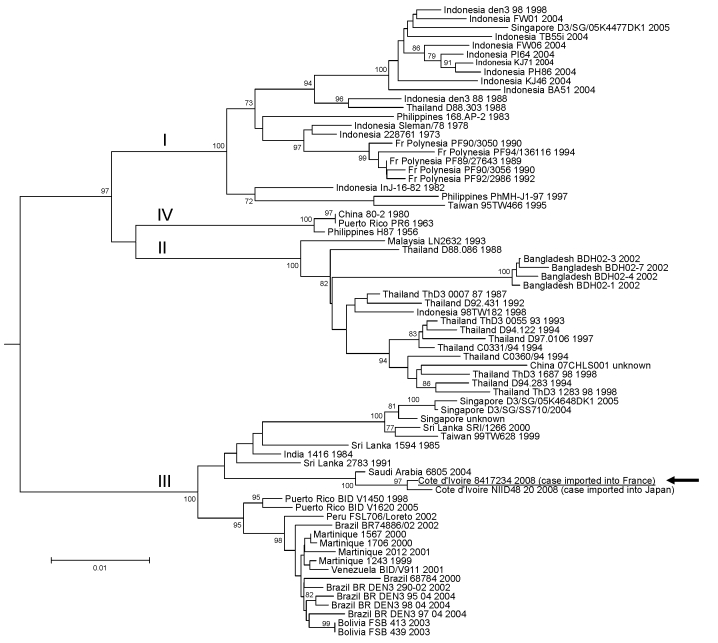
Phylogenetic analysis of selected dengue virus type 3 (DENV-3) sequences. The main genotypes are indicated using roman numerals at the node of the lineage. Sequence identification is as follows: country of origin, strain name, year of isolation/detection. The sequence determined in our study is underlined and designated by an arrow. Phylogenetic studies were conducted by using MEGA version 2.1 (*5*). Genetic distances were calculated with the Kimura 2-parameter method at the nucleotide level. Phylogenetic trees were constructed using the neighbor-joining method. The robustness of the nodes was tested by 500 bootstrap replications. The tree was rooted with DENV-1, DENV-2, and DENV-4 sequences. Scale bar indicates nucleotide substitutions per site.

In Africa, most data on epidemic or endemic dengue activity originate in East Africa. Dengue fever was seldom reported in West Africa. In 2000, DENV-1 was isolated from a French soldier in Côte d’Ivoire (*7*). More recently, an outbreak caused by DENV-2 occurred in Gabon in West Africa (*8*). In this context, it is notable that DENV-3 strains recently caused unexpected outbreaks of dengue hemorrhagic fever in Sri Lanka, East Africa, and Latin America (*9*).

The case presented here demonstrates that epidemics may be undetected or unidentified until diagnosis is assessed in another country from a returning infected visitor, thus drawing attention to an unidentified potential epidemic situation. This situation can be unraveled by a clinician who considers geographic factors in the diagnostic workup and has access to and uses appropriate laboratory capacity to diagnose imported infections. At the time dengue was diagnosed in this patient, cases of yellow fever were reported in the same location of Côte d’Ivoire (Abidjan) (*5*), illustrating concomitant circulation of 2 viruses in which dengue may have remained undetected in the absence of a laboratory-confirmed case in the traveler’s home country. Therefore, this case reinforces the utility of travelers as sentinels for infectious diseases as previously reported (*10*). Our findings reiterate the need for technologic transfer of PCR-based direct diagnostics to reference centers in areas where emergence is likely. These efforts also should embrace serology and encourage close collaboration with world reference centers for confirmation and characterization (*10*).
